# Noninvasive prenatal diagnosis (NIPD) of non-syndromic hearing loss (NSHL) for singleton and twin pregnancies in the first trimester

**DOI:** 10.1186/s13023-025-03558-x

**Published:** 2025-01-27

**Authors:** Huanyun Li, Shaojun Li, Zhenhua Zhao, Lingrong Kong, Xinyu Fu, Jingqi Zhu, Jun Feng, Weiqin Tang, Di Wu, Xiangdong Kong

**Affiliations:** 1 Genetic and Prenatal Diagnosis Center, Department of Obstetrics and Gynecology, The First Affiliated Hospital of Zhengzhou University, Zhengzhou, China; 2 Department of Fetal Medicine and Prenatal Diagnosis Center, Shanghai First Maternity and Infant Hospital, School of Medicine, Tongji University, Shanghai, China; 3 Celula (China) Medical Technology Co., Ltd., Chengdu, China

**Keywords:** Noninvasive prenatal diagnosis, Non-syndromic hearing loss, Haplotype construction, Bayes factors, Twin

## Abstract

**Background:**

Noninvasive prenatal diagnosis (NIPD) has been proven feasible for non-syndromic hearing loss (NSHL) in singleton pregnancies. However, previous research is limited to the second trimester and the application in twin pregnancies is blank. Here we provide a novel algorithmic approach to assess singleton and twin pregnancies in the first trimester.

**Methods:**

A 324.614 kb capture panel was designed to selectively enrich target regions. Parental haplotypes were constructed by target sequencing of blood samples from the parents and the proband. Then single nucleotide polymorphisms (SNP) within target regions were classified into four and six categories in singleton and twin pregnancy, respectively. Combining relative haplotype dosage change (RHDO) and the Bayes factor (BF), fetal fraction (FF) and fetal genotype were deduced in singleton and twin pregnancies. The pregnant women’s NIPD results were validated by invasive prenatal diagnosis and Sanger sequencing.

**Results:**

Sixteen women with singleton pregnancies and one woman with a twin pregnancy were recruited. Among the 16 singleton pregnancies, NIPD was successfully applied in 15 families and the coincidence rate with invasive prenatal diagnosis was 100% (15/15). Only one family NIPD result is “no call” because the imbalance distribution of SNP sites makes it difficult to estimate recombination events. Most (13/15) of pregnant women were diagnosed in the first trimester and the earliest gestation week was the 7th week. The twin pregnancy was a dichorionic diamniotic twin (DCDA). NIPD confirmed one fetus is affected, and another is a carrier with c.299_300delAT of *GJB2* gene.

**Conclusion:**

This study represents the pioneering evidence in the field, demonstrating the feasibility of NIPD for NSHL in twin pregnancies. Moreover, it provides a novel and advanced diagnostic approach for families at high risk of NSHL during pregnancy, offering earlier detection, enhanced safety, and improved accuracy.

**Supplementary Information:**

The online version contains supplementary material available at 10.1186/s13023-025-03558-x.

## Background

Congenital hearing loss is one of the most frequent sensorineural disorders, affecting 1–2 in every 1000 neonates [1]. The epidemiological data revealed that genetic causes account for up to 80% of congenital hearing loss, and most of them (70%) are regarded as non-syndromic hearing loss (NSHL) without other medical anomalies [2, 3]. Hereditary deafness exhibits significant heterogeneity, both clinically and genetically, with over 300 genetic loci and more than 100 causative genes implicated in its pathogenesis [4]. In the Chinese population, the most frequent pathogenic NSHL mutations reside in the *GJB2* gene and *SLC26A4* gene [5, 6]. Current therapeutic interventions for individuals with hearing impairment primarily include hearing aids and cochlear implants. A delayed diagnosis can lead to life-long health issues that could be ameliorated with early intervention and treatment.

For those couples identified as pathogenic *GJB2* gene or *SLC26A4* gene mutation carriers, prenatal diagnosis is an essential way to assess the risk of fertility and guide rehabilitation treatment. Invasive prenatal diagnostic procedures, such as chorionic villus sampling (CVS) and amniocentesis, are widely regarded as the gold standard for genetic diagnosis. However, these methods are associated with a small but significant risk of miscarriage or stillbirth, with reported incidences ranging from 0.1 to 0.3% [7]. In 1997, the discovery of the cell-free fetal DNA (cffDNA) in maternal plasma laid a foundation for non-invasive prenatal diagnosis (NIPD) [8]. Until now, the existing NIPD approaches can be divided into two categories in the diagnosis of NSHL. The first one is relative haplotype dose (RHDO) analysis. Combining high-throughput sequencing of targeted regions and hidden Markov models, Duan et.al realized the first NIPD for a family with *GJB2* gene mutation in 2014 [9]. The second way is relative variant dose (RMD) analysis by circulating single-molecule amplification and resequencing technology (cSMART) [10] or digital PCR [11]. Both cSMART and digital PCR rely on the dosage changes of hotspot mutations between wild-type and mutant alleles to determine the fetal genotype. In that case, RMD requires a relatively higher fetal fraction (FF) and stricter experiment requirements. RHDO does not detect variants itself but infers the inheritance of parental haplotypes by counting the numerous specific SNP alleles dose changes around the pathogenic genes. In this context, RHDO is not limited by the type of mutation and has a wider range of applications. In the context of maternal DNA background noise, the detection of multiple alleles to infer genotypes is more accurate and feasible than directly detecting a single pathogenic variant, especially for recessive hereditary diseases like NSHL.

The earliest detection of non-invasive prenatal testing based on fetal cells is at 8 weeks of gestation [12]. However, the rarity of fetal cells and the complexity of the experimental process limit its clinical application. Furthermore, published reports based on cffDNA were limited to singleton pregnancies during the second trimester, the twin pregnancies and the first-trimester NIPD remain underexplored in NSHL. With the increasing use of ovulation drugs and advancing maternal age, the frequency of twin pregnancies is increasing [13]. Miscarriage risk associated with twin pregnancies is greater when compared to singleton pregnancies, potentially increasing the risk for pregnancy loss if invasive prenatal diagnostic procedures are performed [14].

As such, to develop a comprehensive and accurate NIPD assay for determining fetal NSHL genotypes in at-risk pregnancies, we performed non-invasive twin detection in NSHL with the *GJB2* gene for the first time based on our successful experience in Duchenne muscular dystrophy twin detection [15]. By target sequencing of the *GJB2* gene and *SLC26A4* gene of the trio blood sample, selecting informative SNP, and calculating Bayes factor (BF), the modified prototype assay successfully diagnosed the dichorionic diamniotic twin (DCDA) genotype. Furthermore, this study achieved earlier NIPD for pathogenic *GJB2* gene and *SLC26A4* gene carrier couples, which shows great potential and promise for clinical application.

## Materials and methods

### Sample collection and detection workflow

**Fig. 1:**
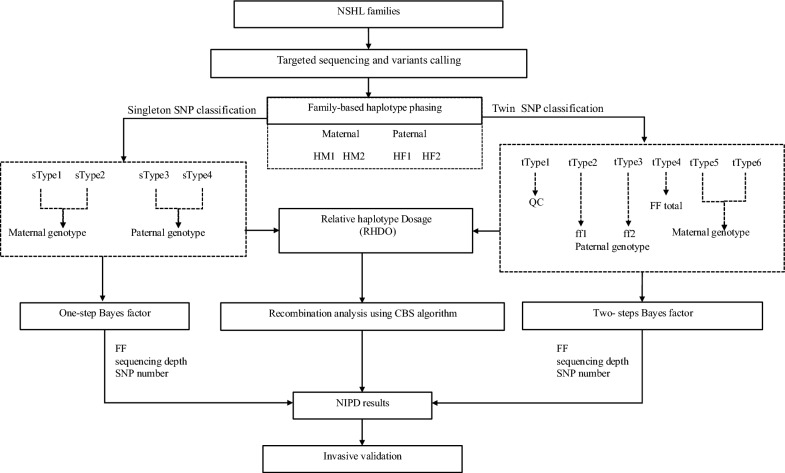
The workflow of NIPD of NSHL families. The NSHL trio family samples were collected and processed after genetic counseling. Then the samples were sequenced on the designed panel and parental haplotypes were identified through trio family sequencing information. The maternal haplotype with the pathogenic mutation was named HM1, and the haplotype with a wide-type allele was named HM2. Similarly for paternal haplotype, HF1 is a pathogenic haplotype. Then the informative SNPs were selected with specific functions as illustrated. RHDO and Bayes factor were performed to identify fetal genotypes inherited from parents. CBS algorithm was used to judge recombination events. Quality control measures, including fetal fraction (FF), sequencing depth, and the number of informative SNPs, were implemented to ensure diagnostic accuracy. All the NIPD results were verified

The study workflow is illustrated in Fig. 1. Seventeen families with pathogenic *GJB2* gene or *SLC26A4* gene mutation were enrolled from March 2021 to October 2023 after genetic counseling and a receipt of informed consent. Sixteen singletons and one twin pregnancy of DCDA were confirmed by ultrasound. For each family, peripheral samples were collected from the pregnant mother (10 ml), father (2 ml), and proband (2 ml). The study was approved by the Ethics Committee of First Affiliated Hospital of Zhengzhou University.

### Library preparation and target sequencing

Genomic DNA (gDNA) was extracted by Nucleic Acid Extraction or Purification Kit (NaHaiTM, China). Subsequently, the gDNA from the trio family (mother, father, and proband) was broken into fragments with an average length of ~ 200 bp by the sonicator (Bioruptor Pico). Maternal plasma was isolated using a two-step centrifugation protocol (See details in Supplementary materials). Then the fragmented gDNA and cfDNA underwent end-repair and added A-tailing. Following barcode ligation, the PCR amplification was performed to enrich the library. A 750 ng library was hybridized with a designed probe panel by incubation at 80 °C for 5 min on a PCR instrument. The target regions were subsequently captured, and the captured library was further amplified via PCR. The amplified libraries were quantified using Qubit3.0 (Invitrogen, Breda, Netherlands) and sequenced on the Ion Proton platform (Thermo Fisher Scientific, Lithuania).

### Targeted sequencing design

**Fig. 2:**
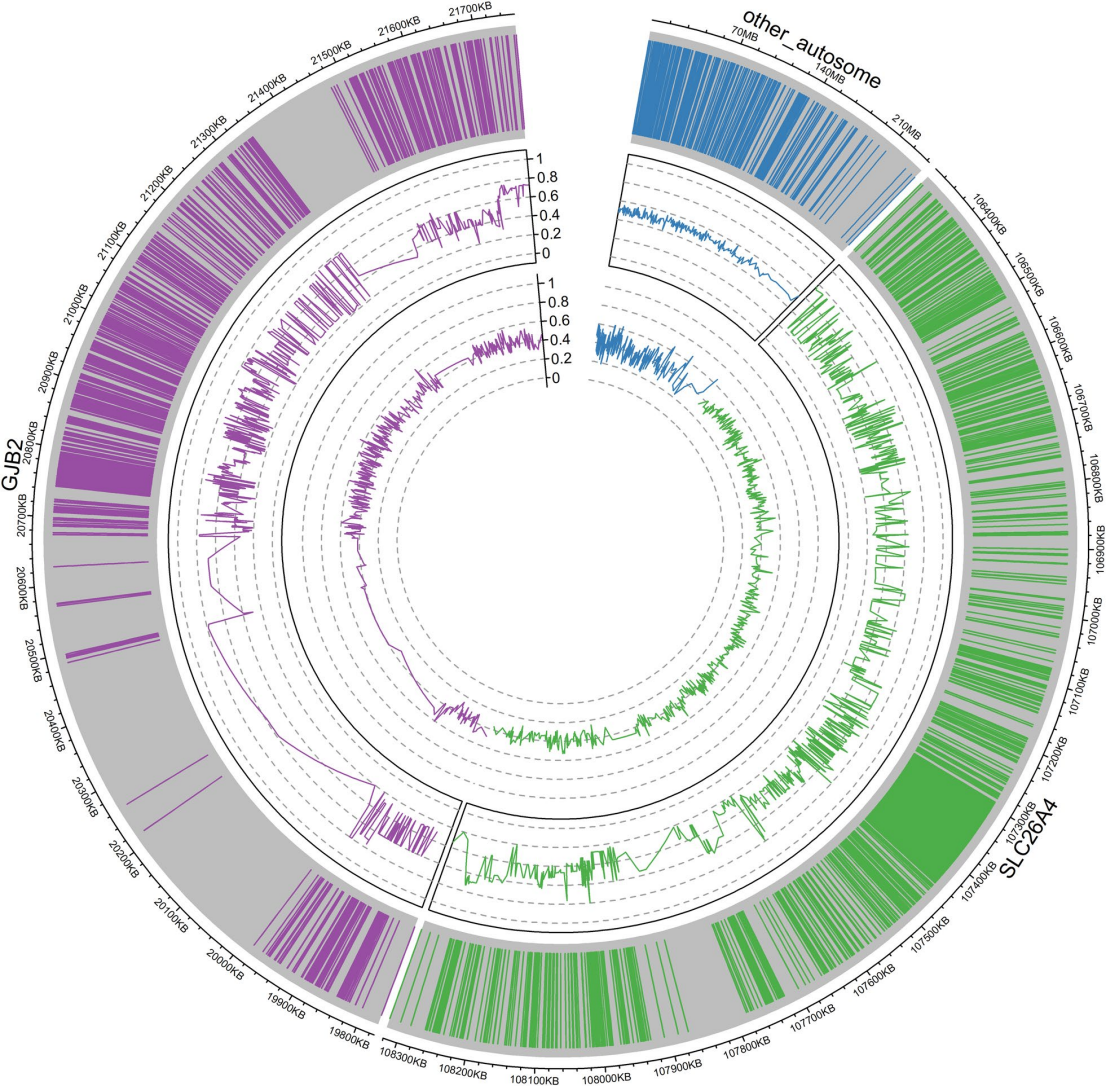
Illustration of the capture panel design. The capture region, SNP site frequency, and GC content were illustrated from outside to inside. Purple and green denote the *SLC26A4* and *GJB2* gene and their spanning 1 Mb regions, respectively. Blue indicates the distribution of 203 SNPs across 1–22 autosomal loci, which were utilized for calculating fetal fraction (FF) and ensuring quality control

A 324.614 kb capture panel TargetSeq® One kit (iGeneTech, China) was designed to selectively enrich target regions based on the reference genome (GRCh37/hg19). The panel covered *GJB2* and *SLC26A4* genes in all exon regions (including untranslated regions), 500 bp intronic regions adjacent to exons, and 10,000 bp upstream or downstream of the target gene. In addition, there were 203 highly heterozygous SNPs (MAF > 0.45) distributed across chromosomes 1–22. (Fig. 2).

### Classification of SNPs and fetal fraction calculation

Haplotype phasing was performed using trio family samples based on Mendel’s law. The maternal pathogenic haplotype was defined as HM1 and the wild-type haplotype was defined as HM2. Similarly, paternal haplotypes were named HF1 and HF2. For singleton pregnancy, Informative SNP sites were those homozygous for one parent and heterozygous for another parent. The sType1 allele would show an imbalance if the fetus inherited HM1 and similarly the sType2 allele would change if the fetus inherited HM2. Paternal inheritance could be judged through sType3 and sType4 SNP sites. The fetal fraction (FF) was calculated via the parent’s homozygous SNP but with different genotypes in maternal plasma (f) by the following equation: f = 2a/(a + b), where “a” is the read depth of the fetal inherited paternal allele and “b” is the read depth of the allele shared by the fetus and pregnant woman.

For twin pregnancy, SNPs were categorized into six categories, named tType 1 to tType 6 (Table 1). tType 1 SNPs served as controls to assess sequencing error rates and detect potential sample cross-contamination. tType 2–4 SNPs were used to infer fetal paternal-inherited haplotype and fetal fraction (named ff1 for the lower fetal fraction, ff2 for the higher fetal fraction, and FFtotal for the total fetal fraction, respectively). tType 5 and 6 SNPs were specifically employed to determine the maternal-inherited haplotypes in each fetus.

**Table 1:** The principle and function of informative SNP classification

SNP type	Father		Mother		Proband		Classification and function of informative SNPs
	HF1	HF2	HM1	HM2	HF1	HM1	
sType1	A	A	A	a	A	A	Father: homozygous Mother: heterozygous SNP allele imbalance: inherit HM1
	a	a	a	A	a	a	
sType2	A	A	a	A	A	a	Father: homozygous Mother: heterozygous SNP allele imbalance: inherit HM2
	a	a	A	a	a	A	
sType3	A	a	a	a	A	a	Father: heterozygous Mother: homozygous New SNP allele: inherit HF1
	a	A	A	A	a	A	
sType4	A	a	A	A	A	A	Father: heterozygous Mother: homozygous New SNP allele: inherit HF2
	a	A	a	a	a	a	
tType1	A	A	A	A	A	A	Father: homozygous Mother: homozygous QC
	a	a	a	a	a	a	
tType2	a	A	A	A	a	A	Father: heterozygous Mother: homozygous New SNP allele: inherit HF1; ff1
	A	a	a	a	A	a	
tType3	A	a	A	A	A	A	Father: heterozygous Mother: homozygous New SNP allele: inherit HF2; ff2
	a	A	a	a	a	a	
tType4	a	a	A	A	a	A	Father: homozygous Mother: homozygous FFtotal
	A	A	a	a	A	a	
tType5	A	A	A	a	A	A	Father: homozygous Mother: heterozygous SNP allele imbalance: inherit HM1
	a	a	a	A	a	a	
tTpye6	a	a	A	a	a	A	Father: homozygous Mother: heterozygous SNP imbalance: inherit HM2
	A	A	a	A	A	a	

### Haplotype analysis

The allele frequencies of informative SNPs were used to quantify the dosage changes of the pathogenic haplotype and the wild-type haplotype. Based on allele frequency imbalance, the probability of fetal inherited pathogenic or wild-type haplotypes was estimated using the BF, as described in prior methodologies [16]. For singleton pregnancy, if a BF value ≥ 10 indicated that the fetus inherited the HF1/HM1 haplotypes, while a BF value of ≤ 0.1 suggested inheritance of the HF2/HM2 haplotypes. In cases where the BF fell between 0.1 and 10, the NIPD result was classified as “no call”. For twin pregnancy, the maternal‐inherited haplotype was deduced using RHDO through a two‐step Bayes factor approach, as we described before [15]. The first step was to determine if the twins inherited the same maternal haplotype. In the second step, the inherited maternal haplotype for each fetus was deduced based on the decision of the first step. Bayes factor was calculated at each step (BF1 for step 1 and BF2 for step 2) by dividing the likelihood of obtaining the observed difference of RHDO between tType 5 and tType 6 SNPs under two opposite hypotheses. Paternal inheritance was deduced by the dose change of tType2 and tType3. Besides, all the fetal haplotype speculations were tested by the CBS algorithm to exclude the influence of recombination events on the NIPD results.

### Invasive prenatal diagnosis

The twin underwent a double separate amniocentesis and the other fifteen singleton pregnant women also underwent amniocentesis at 18–24 weeks of gestation. In one case, a cerclage of the cervix during the second trimester rendered invasive prenatal diagnosis unsuitable; therefore, a neonatal sample was collected after birth for genetic analysis. All amniotic fluid samples and neonatal samples were subjected to Sanger sequencing to validate the accuracy of the NIPD.

## Results

### Trio family information

A total of 17 pregnant women were recruited. Among these, 14 singleton pregnant women provided blood samples in the first trimester, from 7 to 12^+5^ weeks. Only two singleton pregnant women had blood collected after 13 weeks. One woman provided a blood sample at 19^+1^ to test panel feasibility and another pregnant woman underwent blood collection at 28 weeks due to a recent cerclage of the cervix procedure, which was performed to prevent miscarriage and precluded the option of amniocentesis. The twin pregnant woman had NIPD at 17^+3^ weeks. Among the 17 families, 7 families had a proband with *GJB2* gene mutations, while the remaining 10 families with *SLC26A4* gene mutations (see details in supplementary table).

### NIPD results

#### Sequencing information

The prepared gDNA and cfDNA of 17 families were sequenced by target region capture, and the average of total reads is 3,245,415 (957,564–7,114,056). The average sequencing depth of each sample range from 62x to 746x (average: 273x) and the ratio of more than 300x ranges from 25.36% to 87.56% (average: 50.90%). The singleton families’ informative SNPs range from 8 to 196 for sType1 and sType2, 8 to 178 for sType3 and sType4. The twin family SNP is 50, 46, 57, 70, 15, 86 from tType1 to tType6, respectively.

#### Sixteen singleton NIPD result

NIPD was successfully applied in fifteen families. Among these cases, 3 fetuses were identified as affected, 4 as carriers, and 8 as unaffected. Notably, recombination events were detected in two fetuses (P15, P17). However, the breakpoints of these recombination events, as estimated by the circular binary segmentation (CBS) algorithm, were located at a considerable distance from the mutation site, ensuring that the NIPD results remained unaffected and reliable (Fig. S1). Only one failed family (P6) yielded inconclusive results due to an imbalanced distribution of SNP sites, with the majority located downstream of the maternal pathogenic variant (Fig. S2). In this case, the presence of potential recombination events could not be determined, leading to a “no call” result for the maternal haplotype.

#### One twin NIPD results

For the twin pregnancy parents, the mother is a *GJB2* gene carrier of the c.299_300delAT mutation, and the father is a *GJB2* gene carrier of the c.235delC mutation. From the informative SNP scattered in the *SLC26A4* gene panel, the twin’s zygosity could be deduced. tType2 and tType3 elevated in both *SCL26A4* gene and *GJB2* gene panel, which means the twin inherited HF1 and HF2, respectively. It proved the twin was a fraternal twin and the fetal fraction of the two fetuses is similar, about 6%. Correspondently, we could see clearly that FFtotal is about 12% from tType4 SNPs. The dosage changes of the *GJB2* gene tType5 and tType6 SNPs combined with the two-step Bayes factor analysis, demonstrated that both fetuses inherited pathogenic maternal haplotype (HM1) simultaneously. For this *GJB2* mutation carrier twin family, one fetus was identified as affected, while the other was determined to be a carrier of the *GJB2* c.299_300delAT mutation.

### Validation of NIPD results

16 pregnant women underwent invasive prenatal diagnosis, including the case with “no call” result. Among the successful NIPD cases, there is a woman who had a cerclage of cervix procedure, the fetus underwent peripheral blood Sanger sequencing after birth. The results showed that the accuracy of singleton pregnancies NIPD was 100% (15/15). For the twin, the double separate amniocentesis and Sanger sequencing results are also coordinated with NIPD (Fig. S3).

## Discussion

Appropriate prenatal diagnosis of hearing loss could give carrier couples more options for future family planning and probably the preparation for the health and educational needs of the affected neonates [17]. In this study, which focused on NIPD of NSHL, the earliest gestational age at which testing was successfully performed was 7 weeks. Among the sixteen singleton pregnant women, NIPD was successfully applied in 93.75% (15/16) of families and the coincidence rate with invasive prenatal diagnosis was 100% (15/15). Only one NIPD result is “no call” because the imbalance distribution of SNP sites makes it difficult to estimate recombination events. Most (13/15) of pregnant women were in the first trimester and the earliest gestation week was the 7th week.

Besides, due to the wide application of reproductive technology, the probability of multiple pregnancies is increasing. The singleton NIPD algorithms may yield to inaccurate results in dizygotic twins since the fetal fraction of the affected fetus could be lower and result in a dosage change not as considerable as expected. To address this, we proposed a two‐step Bayes factor with the first step to distinguish whether the twins inherit different haplotypes. The second step could indicate whether the pathogenic haplotype was inherited for every fetus. Importantly, if the first step indicates the twin inherited identical haplotypes, only a single puncture operation may be required for invasive confirmation, thereby minimizing the risk of miscarriage.

Whether singleton or twin pregnancy, genomic DNA target sequencing requires no complicated experimental procedure, such as the previously reported haplotype-assisted methods, and is cost-effective if the appropriate array is designed. Moreover, the turnaround time, including the sampling process and sequencing on the Ion Proton platform, can be as short as 1 week. Furthermore, bioinformatics analysis can be finalized in as little as 1 day, making this approach highly suitable for large-scale clinical implementation.

Despite its advantages, several limitations were identified, and corresponding solutions were implemented to ensure the accuracy of NIPD. First, the traditional proband-based haplotype requires a complete trio family to construct the parent haplotype. However, no proband is also available in our study design. Families with a previous reproductive history, whether involving normal offspring or carriers, can be used to construct haplotypes [9]. For families without reproductive history, haplotypes can be inferred through grandparents. Second, the NIPD results might be disturbed by recombination events. The CBS algorithm could predict the recombination event, which is used to estimate copy number variation (CNV) data and identify the reasonable breakpoint [18]. Researchers can then assess whether the recombination break point affected the identification of pathogenic variants. Third, consanguineous marriages may lead to extended regions of homozygosity, reducing the number of informative SNPs and rendering this method unsuitable for such cases.

**Fig. 3:**
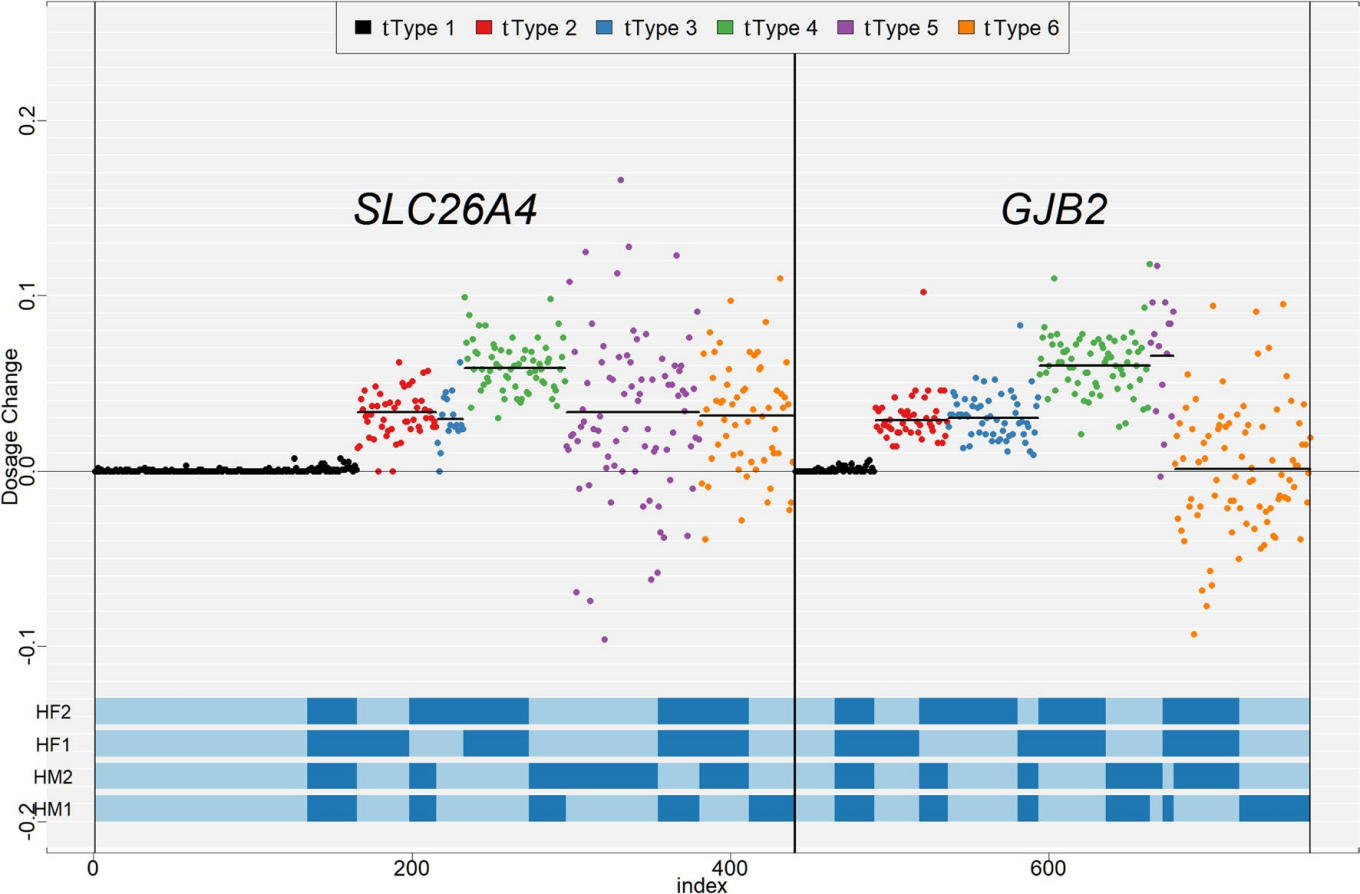
SNP classification and dosage change. The horizontal coordinates represent SNP sites and are sorted by SNP type and genome coordinates, with SNP types color-coded. The vertical axis represents the dose change for each SNP site, and the black horizontal line represents the mean dose change for each SNP type. The parent haplotype is at the bottom, with light blue representing the reference base and dark blue representing the variant base

For the twin pregnancy with *GJB2* gene mutation, the twin’s fetal fraction is coincidentally almost identical. Luckily, the *GJB2* gene mutation inheritance is diagnosed clearly in this case. However, this scenario would be more complex for families carrying *SLC26A4* gene mutations. For the* SLC26A4* gene, the twins inherited four parents’ haplotypes (Fig. 3). When the fetal fractions of the twins are identical and all four haplotypes are inherited, two possible inheritance scenarios arise: (1) both fetuses could be carriers of the pathogenic mutation, or (2) one fetus could be affected while the other is unaffected. In such cases, invasive prenatal diagnosis becomes essential to resolve the ambiguity and provide a definitive diagnosis.

## Conclusion

Prenatal diagnosis is an important step for couples with an established pregnancy at risk for NSHL to determine at an early stage whether their fetus is affected by a sensory disability. This information allows couples to assess reproductive risk and make informed decisions. If the pregnancy is continued, clinicians can better prepare to manage and treat the condition from birth. Conversely, if the couple chooses to terminate the pregnancy, a first-trimester diagnosis permits medical abortion, reducing the physical and emotional trauma associated with more surgical procedures [19]. Furthermore, our algorithm also proved the NIPD efficiency of monogenic disorders in dizygotic twin pregnancies. The availability of a reliable and accurate NIPD genotyping method offers a safer and more convenient prenatal option, minimizing risks to both the mother and fetus compared to invasive diagnostic procedures.

## Supplementary Information

Supplementary material 1.

Supplementary material 2.

## Data Availability

The datasets for this article are not publicly available due to concerns regarding participant/patient anonymity. The datasets used during the current study are only available from the corresponding author on reasonable request.

## Abbreviations

NIPD: Noninvasive prenatal diagnosis
NSHL: Non-syndromic hearing loss
SNP: Single nucleotide polymorphisms
RHDO: Relative haplotype dosage
BF: Bayes factor
FF: Fetal fraction
DCDA: Dichorionic diamniotic twin
CVS: Chorionic villus sampling
cffDNA: Cell-free fetal DNA
RMD: Relative variant dose
cSMART: Circulating single-molecule amplification and resequencing technology
CNV: Copy number variation
