# Supplementary material for: Noninvasive prenatal diagnosis (NIPD) of non-syndromic hearing loss (NSHL) for singleton and twin pregnancies in the first trimester
Source: Orphanet J Rare Dis. 2025 Jan 27;20:40. doi: 10.1186/s13023-025-03558-x (PMC11773923; doi:10.1186/s13023-025-03558-x)
Supplement: Supplementary file 2 — Supplementary material 2. [file 13023_2025_3558_MOESM2_ESM.docx]

**Supplementary method**

**DNA extraction**

Maternal plasma was separated by initial centrifugation at 2000×g for 20 min, followed by second centrifugation at 4,000×g for 20 min. Cell-free DNA (cfDNA) was extracted from 2mL of plasma immediately after plasma isolation using the Nucleic Acid Extraction or Purification Kit (NaHai^TM^, China). The genomic DNA (gDNA) of each family member was extracted directly from 40μL of blood using the same kit mentioned above.

**Library preparation**

The genomic DNA of each family member was fragmented into an average length of about 200 bp using restriction endonuclease. The pregnant cfDNA and fragmented genomic DNA were end-repaired and ligated to barcoded adapters, followed by 9 and 7 cycles of polymerase chain reaction (PCR), respectively. Hybridization-based DNA capture was performed with 750ng DNA input using a combined 324.614kb TargetSeq® One capture panel (iGeneTech, China). After incubation at 50°C for 18 hours, DNA sequences bound to probes were recovered by streptavidin-coated magnetic beads and then were posed another 12 cycles of PCR. Target-enriched DNA libraries were quantified by Qubit 3.0 (Invitrogen, Breda, Netherlands) and mixed before sequencing. Target-enriched DNA libraries were sequenced on the Ion Proton platform (Thermo Fisher Scientific, Lithuania).

**Variant calling and Quality control**

Sequencing reads were mapped to the human reference genome (hg19) using the TMAP software (version 5.2.25) with the ‘Mark as Duplicate Reads’ option selected. PCR duplicates and multiple aligned reads were removed. For gDNA samples, small variants were called by the Torrent Variant Caller software (version 5.2.25) using default parameters. Only small variants with sequencing depth greater than 30x and quality scores greater than 50 were retained for haplotype construction. For the pregnancy cfDNA sample, allele frequency was calculated for each SNP locus. Loci with a sequencing depth less than 100 or an error rate (defined as the frequency of indel) greater than 0.01 were removed to minimize sampling and sequencing errors. The quality control of the fetal fraction was at 1% in singleton and twin pregnancies, as we illustrated before. gDNA sequence depth and cfDNA depth set quality control in 30x and 150x, respectively. For the informative SNP used to determine maternal inheritance (sType1, sType2, tType5,tType6), the least number is 10. For the informative SNP used to determine maternal inheritance (sType3, sType4, tType2,tType3), the least number is 5.

**Figure S1. The two recombination families.**

**Figure S2. The failed P6 family.**


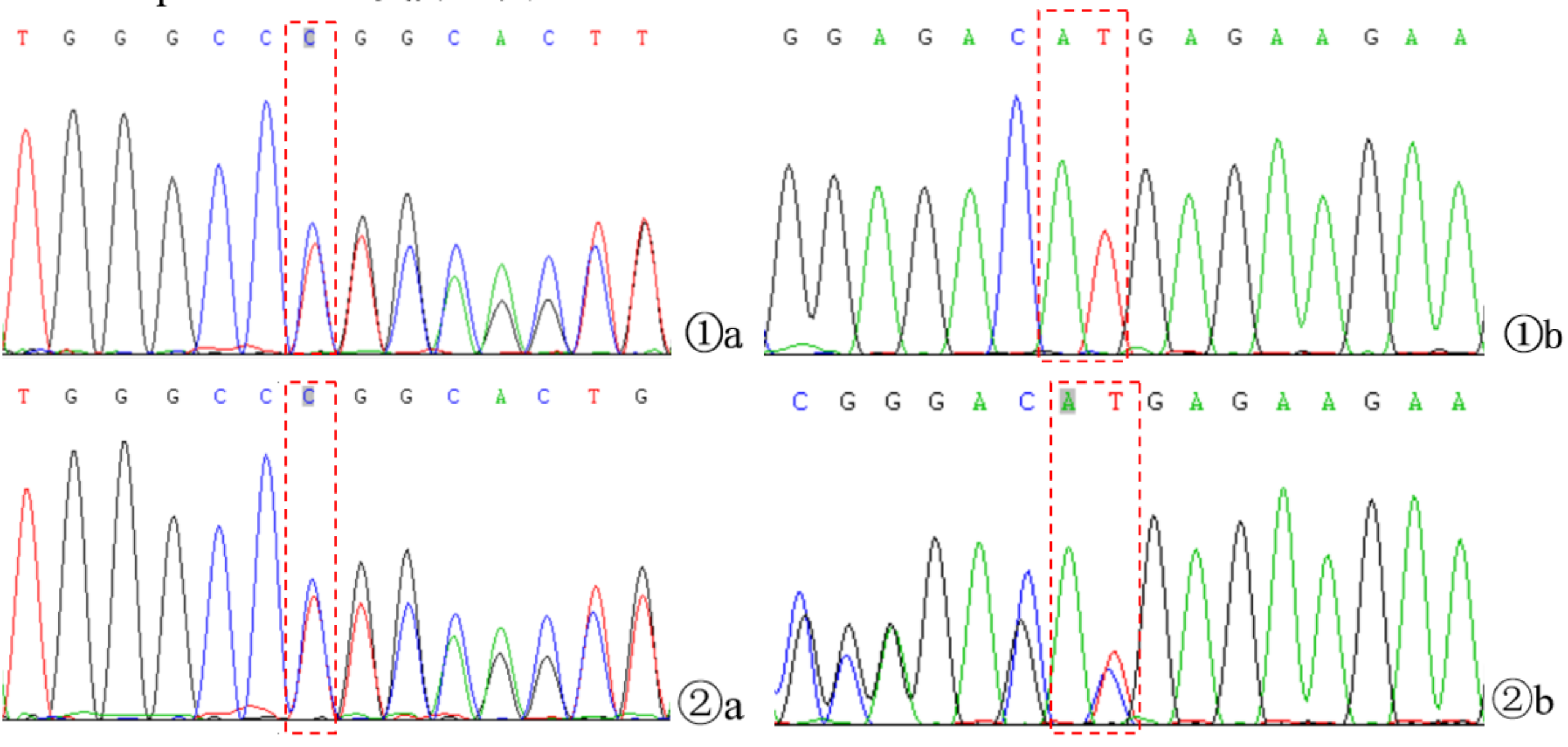


**Figure S3. The double separate amniocentesis and Sanger sequencing results.**
